# Likelihood of breast cancer among women presenting with breast-related symptoms in primary care, with a focus on isolated mastalgia: a retrospective cohort study

**DOI:** 10.1186/s12875-026-03357-8

**Published:** 2026-05-08

**Authors:** Simon K. L. van Ieperen, Floris A. van de Laar, Reinier P. Akkermans, Annemarie A. Uijen

**Affiliations:** Department of Primary and Community Care, Radboud University Medical Centre Nijmegen, PO Box 9101, Geert Grooteplein Noord 21, Nijmegen, 6500 HB The Netherlands

**Keywords:** General practice, Primary care, Mastalgia, Breast neoplasms, Predictive value

## Abstract

**Background:**

Breast-related symptoms are common in primary care and frequently prompt imaging or referral. While a palpable breast lump is strongly associated with breast cancer, isolated mastalgia is generally considered low risk. Most evidence comes from secondary care, limiting applicability to general practice.

**Aim:**

To estimate the likelihood of breast cancer associated with breast-related symptoms in primary care and to evaluate current GP management of women with isolated mastalgia.

**Design and setting:**

Retrospective cohort study using electronic health records from a Dutch primary care research network.

**Methods:**

We included women aged ≥ 30 years presenting with breast-related symptoms or diagnosed with breast cancer between 2014 and 2021. Multivariable logistic regression assessed associations between symptoms and breast cancer. Mastalgia was classified as either isolated (without any other breast-related symptoms) or in combination with other symptoms. In women with isolated mastalgia, GP-initiated imaging and referrals were recorded and evaluated.

**Results:**

We included 2073 episodes from 1752 women (median age: 45.0 years, range 30–100 years); 189 episodes (9.1%) resulted in breast cancer. Presentation with a breast lump (OR 10.57, 95%-CI 4.89–22.84), skin or nipple retraction (OR 5.54, 95%-CI 2.28–13.48), and increasing age (OR 1.07 per year, 95%-CI 1.05–1.08) were independently associated with breast cancer. Mastalgia was associated with lower odds (OR 0.56, 95%-CI 0.34–0.92). Mastalgia was reported in 26 episodes that ultimately resulted in a breast cancer episode, but no cases occurred among women with isolated mastalgia (0 of 588 episodes). Despite this, 39.6% of women with isolated mastalgia underwent breast imaging, nearly half within three months.

**Conclusion:**

In primary care, breast cancer is strongly associated with a palpable lump, skin or nipple retraction, and increasing age. Isolated mastalgia was not associated with breast cancer. However, mastalgia occurring in combination with other breast-related symptoms may indicate a higher risk. Imaging and referral may often be safely deferred in women presenting with short-duration isolated mastalgia.

## Introduction

Breast cancer is the most frequently diagnosed cancer among women worldwide and a leading cause of cancer-related morbidity and mortality. Worldwide, an estimated 2.3 million new cases of breast cancer were diagnosed in women in 2022, with projections suggesting a 38% increase by 2050 [1]. In the Netherlands, approximately 18,000 women are diagnosed annually, with diagnostic pathways typically initiated either through population-based screening or presentation with symptoms in primary care [2]. The Dutch population-based breast cancer screening program invites women aged 50–75 years for biennial mammography. General practitioners (GPs) therefore play a pivotal role in the early assessment, reassurance, and referral of women with breast-related complaints.

Breast-related symptoms are common in general practice, yet only a minority of symptomatic women are ultimately diagnosed with breast cancer. A palpable breast lump is consistently reported as the most predictive symptom, whereas isolated mastalgia is generally considered to have a very low association with malignancy [3–7]. Previous research showed that women consulting their GP for mastalgia have a 0.9% risk of breast cancer, whereas a breast lump accounts for an 8.1% risk of breast cancer [8]. A more recent study conducted in an imaging center demonstrated a risk of 0.4% for mastalgia, lower than the incidence of breast cancer in the screening population, opposed to 5.4% for women referred for a lump [6]. So, the added value of imaging in patients presenting solely with mastalgia seems to be low [5, 9, 10]. 

Nevertheless, mastalgia often leads to diagnostic imaging or referral, potentially contributing to overdiagnosis, patient anxiety, and increased healthcare costs [11, 12]. 

Previous studies estimating the likelihood of breast cancer by presenting symptom have important limitations for primary care practice. Several were conducted in secondary care or imaging settings, where patient selection differs substantially from general practice [4–6, 9, 10]. 

Others used historical data that may not reflect current population characteristics, diagnostic pathways, or guideline recommendations [9]. Additionally, a more recent study set in primary care identified patients retrospectively based on a final diagnosis of breast cancer, rather than prospectively from the initial presenting symptom, limiting their applicability to real-time clinical decision-making [3]. 

Contemporary, symptom-based estimates of breast cancer likelihood in primary care are therefore needed to support evidence-based reassurance and referral decisions. In particular, clarity regarding the clinical significance of isolated mastalgia is essential, given ongoing uncertainty and variation in GP management.

This study aimed to estimate the likelihood of breast cancer associated with initial breast-related symptoms in primary care using a large population-based dataset. Additionally, we examined diagnostic imaging and referral patterns among women presenting with isolated mastalgia, to assess current practice in relation to guideline recommendations.

## Methods

### Study design and data source

We conducted a population-based cohort study using routinely collected electronic health record data from the Family Medicine Network (FaMe-Net), a long-standing Dutch practice-based research network affiliated with Radboud University Medical Center, Nijmegen, the Netherlands [13, 14]. This network includes around 40,000 patients (32 GPs, 7 general practices) and registers all patient encounters since 1971.

GPs routinely code each episode of care. An episode of care is defined as a health problem in an individual from the first encounter until the most recent or final encounter. For all encounters within an episode of care, the GP registers the patient’s reason for encounter (RFE), GP’s diagnosis and all performed diagnostic and therapeutic interventions (e.g. physical examination, radiologic imaging (including mammography and ultrasound) and referrals) according to the World Health Organisation (WHO) International Classification of Primary Care, 2nd edition (ICPC-2) [15]. Each episode is assigned an ICPC code reflecting the GP’s working diagnosis, which is continuously updated as new insights arise (for example the diagnosis can change from ‘breast lump’ to ‘breast cancer’ after imaging results). In the remainder of this article, an episode of care is referred to as an ‘episode’. Multiple complaints recorded at the same consultation are linked to separate episodes only if considered distinct health problems. Complaints judged by the GP to belong to the same underlying problem are linked within a single episode. For recurrent breast-related symptoms, the GP determines whether a subsequent consultation is part of the same episode or represents a new episode.

To improve the quality of registration, participating GPs meet regularly to discuss registration and diagnostic criteria. Moreover, the system warns the GP in case of error or inconsistency in registration, resulting in reliable registration.

In the Netherlands, GPs act as gatekeepers to secondary care. Patients with breast-related complaints typically first consult their GP, who performs the initial assessment and determines whether diagnostic imaging (mammography and/or ultrasound) can be requested directly or whether referral to secondary care is indicated. Unlike some healthcare systems, Dutch GPs have direct access to breast imaging without mandatory prior specialist referral.

### Study population

We included all female patients aged 30 years or older who consulted their GP between January 1, 2014 and July 1, 2021 with a breast-related reason for encounter or diagnosis at the first consultation of an episode, defined by the following ICPC-2 codes: breast pain (X18), breast lump/mass (X19), nipple symptom/complaint (X20), other breast symptom/complaint (X21), concern about breast appearance (X22), fear of breast cancer (X26), malignant neoplasm breast (X76), benign neoplasm breast (X79) or fibrocystic disease breast (X88). Additionally, we included all patients with a final diagnosis of breast cancer (X76), regardless of the initial RFE. This allowed us to identify cases in which the initial RFE was coded using a non-breast-related ICPC code. Based on these findings, we decided whether additional RFEs should be considered for inclusion in our study.

Patients were required to have at least six months of follow-up data after the start of the episode, to ensure completion of the diagnostic process. While patients may return after six months with complaints belonging to the same episode, it is likely that the diagnostic process and the final diagnosis are completed within the first six months, making this timeframe sufficient for study purposes. Exclusion criteria were male patients, pregnancy or (recent) breastfeeding, transgender patients (due to different baseline breast cancer risk [16]), inaccessible medical records, erroneous registrations and episodes predating the inclusion period.

### Data extraction

We extracted the following data: year of birth, ICPC-coded RFEs and diagnoses, and all GP-initiated diagnostic and therapeutic interventions within each episode.

To improve clinical specificity, a detailed manual review of electronic medical records was conducted for all included episodes. We extracted all symptoms mentioned in the first consultation of the episode, symptom duration, and, when breast pain was reported, whether the pain was described as localized or diffuse. Localized mastalgia was defined as pain confined to a single focal area, while diffuse mastalgia included bilateral pain, pain in multiple quadrants, or generalized breast discomfort. Findings from physical examination were not included in the symptom classification. Mastalgia was further categorized as isolated (breast pain without any other breast-related symptoms at first presentation) or mastalgia in combination with other symptoms.

If multiple episodes from the same patient appeared to represent a single clinical episode, these were merged after record review. The manual review was performed by one researcher (SvI), with a random sample of 50 records independently checked by a second researcher (AU). Ambiguous cases were discussed with a third investigator (FvdL) until consensus was reached.

### Statistical analyses

Descriptive statistics were used to summarize patient and episode characteristics. Continuous variables were reported as means with standard deviations or medians with interquartile ranges, as appropriate. Categorical variables were summarized using counts and percentages. Patient’s age was calculated using the start date of the episode and the patient’s year of birth. Patients were divided into the age groups 30–49, 50–75 and *≥* 76 years. The age group 50–75 matches the Dutch breast cancer screening program [17]. Incidence rates of breast-related presentations were calculated by dividing the number of episodes starting with a breast-related complaint by the total at-risk female patient population from January 2014 to July 2021 of the included practices, expressed as episodes per 1000 patient-years. The denominator consisted of all female patients aged ≥ 30 years registered in the participating practices during the study period. In total, 16,896 unique female patients aged ≥ 30 years were registered in the participating practices during the study period. The study population was dynamic, allowing patients to enter and leave over time. Patient-years were calculated at the individual level, from the start of registration in the practice or January 1, 2014 (whichever came later) until deregistration, death, or July 1, 2021 (whichever came first), and summed across all individuals. The total number of person-years contributing to the analysis was 70,200. Incidence was calculated for the abovementioned age groups.

Associations between presenting symptoms and a final diagnosis of breast cancer were assessed using multivariable logistic regression. Independent variables included patient age and the presence of the following symptoms at first presentation: mastalgia, breast lump, nipple or skin retraction, nipple discharge, erythema in the breast region, and itching of the breast. A stepwise backward elimination procedure was applied, sequentially removing the least significant variable until only variables with a two-sided *p*-value < 0.05 remained in the final model. Results are reported as odds ratios (ORs) with 95% confidence intervals (CIs).

In a secondary analysis, we focused on women presenting with isolated mastalgia, defined as breast pain in the absence of any other breast-related symptoms at the first consultation. For this subgroup, we described the proportion of episodes in which GPs initiated diagnostic imaging and/or referral to secondary care, as well as the timing of these interventions relative to symptom onset.

All analyses were performed using SPSS version 27.0. A two-sided *p*-value < 0.05 was considered statistically significant.

## Results

**Fig. 1:**
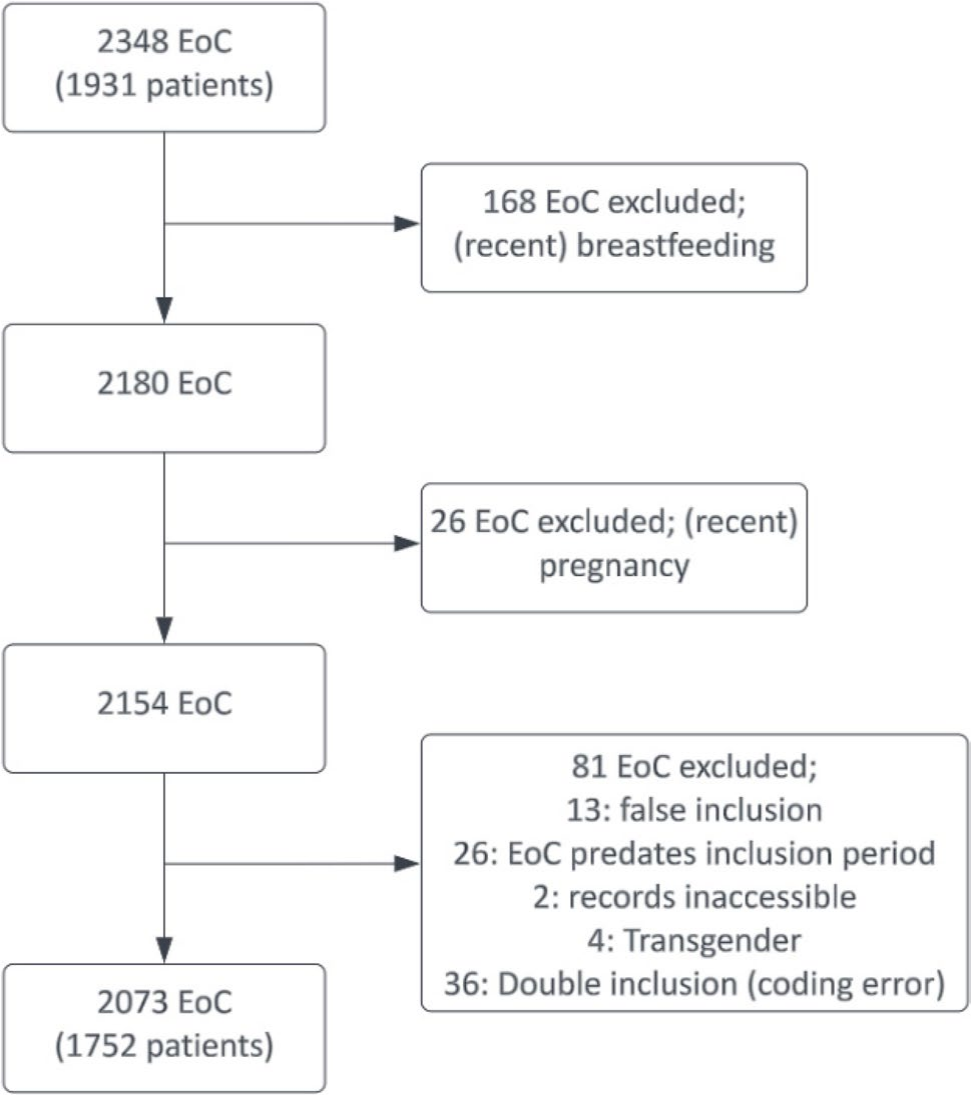
Inclusion of episodes of care and patients

In total, 2348 episodes were analyzed. We finally included 2073 episodes from 1752 patients (Fig. 1). The median age of included women was 45.0 years, range 30–100 years. Of the 2073 episodes, 706 (34.1%) occurred in women aged 50 years or older. Most exclusions were related to pregnancy or recent breastfeeding. Among the 189 episodes with a final diagnosis of breast cancer, four episodes (2.1%) had an initial reason for encounter outside the predefined breast-related ICPC codes: arm complaints (L09, *n* = 1), localized skin swelling or lump (S04, *n* = 2), and local rash or exanthema (S06, *n* = 1). Because these numbers were very small, we did not include these RFE’s for further analysis.

The overall incidence of breast-related presentations in this population was 27.6 episodes per 1000 patient-years (Table 1). Incidence was highest in women aged 30–49 years (38.7 per 1000 patient-years), followed by 50–75 years (18.4 per 1000) and ≥ 76 years (9.4 per 1000).

**Table 1:** Incidence of breast-related complaints in women *≥* 30 years of age

Age	Breast complaints	Patient years (x1000)	Incidence (per 1000 patient years)
30–49	1345	34.8	38.7
50–75	535	29.0	18.4
76+	60	6.4	9.4
Total	1940	70.2	27.6

### Patient characteristics

Table 2 shows the characteristics of included episodes with and without a final breast cancer diagnosis. Women with breast cancer were older (59.5 years) than women without breast cancer (45.8 years, *p* < 0.001). Among the breast cancer episodes, 70 (37.0%) were first detected through the population-based screening program. An additional 20 episodes were initiated during routine screening but resulted in another breast-related diagnosis, such as fibrocystic breast disease. The most frequently reported symptoms at first presentation were mastalgia (*n* = 1011, 48.8%) and a palpable breast lump (*n* = 993, 47.9%).

**Table 2:** Characteristics of the included episodes

	Episodes not resulting in breast cancer	Episodes resulting in breast cancer	*P*-value for group difference
Number of episodes	1884	189	
Age (*n*, %)			
30–49	1320 (70.1%)	47 (24.9%)	
50–75	521 (27.7%)	119 (63.0%)	
76+	43 (2.3%)	23 (12.2%)	
Patient age (years)			
Mean (SD)	45.8 (11.4)	59.5 (14.5)	< 0.001
Symptoms (*n*, %)			
Mastalgia	985 (52.3%)	26 (13.8%)	< 0.001
* Diffuse*	*317 (16.8%)*	*2 (1.1%)*	
* Local*	*446 (23.7%)*	*20 (10.6%)*	
* Unclear*	*222 (11.8%)*	*4 (2.1%)*	
Lump/palpable mass	904 (48.0%)	89 (47.1%)	0.585
Nipple discharge	77 (4.1%)	3 (1.6%)	< 0.001
Skin or nipple retraction	52 (2.8%)	11 (5.8%)	< 0.001
Erythema in breast region	38 (2.0%)	2 (1.1%)	0.066
Itch in breast region	22 (1.2%)	1 (0.5%)	0.108
Isolated mastalgia (*n*, %)	588 (31.2%)	0 (0%)	
Diffuse	230 (12.2%)	0 (0%)	
Local	178 (9.4%)	0 (0%)	
Unclear	180 (9.6%)	0 (0%)	
Screening program (*n*, %)	20 (1.1%)	70 (37.0%)	< 0.001

### Isolated mastalgia: likelihood of breast cancer and GP management

Mastalgia was reported in 26 episodes that ultimately resulted in a breast cancer diagnosis (Table 2). However, when considering isolated mastalgia (breast pain without any other breast-related symptoms at first presentation) no breast cancers occurred (0 of 588 episodes).

**Fig. 2:**
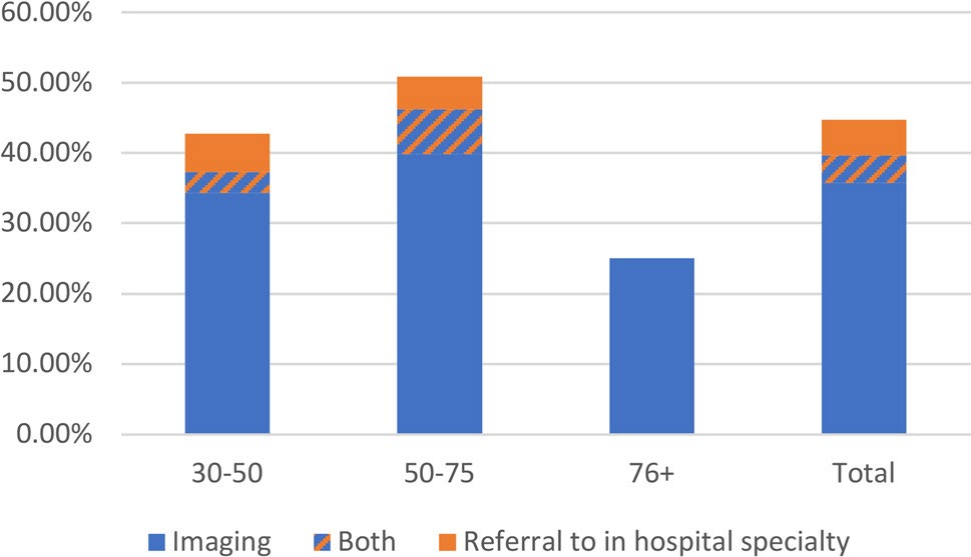
Imaging and referral percentage in the isolated mastalgia group. Percentages are calculated per age group and do not sum to 100%, as not all women underwent imaging or referral during the episode

Among the 588 episodes of isolated mastalgia, GPs initiated breast imaging in 233 (39.6%) episodes and referred patients to secondary care in 53 (9.0%) episodes. Within these groups, both imaging and referral was performed in 23 (3.9%) episodes (Fig. 2). As the current Dutch guideline for breast cancer does not recommend imaging for isolated mastalgia of less than three months’ duration [17], we assessed how often imaging was performed within this period. Among women with isolated mastalgia who underwent imaging (*n* = 233), 116 (49.8%) women received imaging within three months of symptom onset. Localized mastalgia was more likely to prompt imaging in women who underwent imaging within three months (*n* = 42, 43.8%) when compared to diffuse mastalgia (*n* = 36, 33.0%, *p* < 0.001).

In episodes where isolated mastalgia lasted less than three months but imaging or referral was still performed, GPs documented an abnormality on physical examination in 35% (*n* = 41) of cases, most commonly described as ‘firm glandular tissue’, ‘glandular package’, or ‘dubious lump’.

### Factors associated with breast cancer

Table 3 shows the results of the multivariable logistic regression analysis. A breast lump/palpable mass (OR 10.57, 95% CI 4.89–22.84, *p* < 0.001) and skin or nipple retraction (OR 5.54, 95% CI 2.28–13.48, *p* < 0.001) were associated with substantially higher odds of breast cancer, while mastalgia (OR 0.56, 95% CI 0.34–0.92, *p* = 0.02) was associated with lower odds. Increasing age (OR 1.07 per year, 95% CI 1.05–1.08, *p* < 0.001) was also independently associated with higher likelihood of breast cancer.

**Table 3:** Relation between patient’s initial complaints and breast cancer

	Univariate			Multivariate		
	Odds ratio	95% confidence interval	*P*-value	Odds ratio	95% confidence interval	*P*-value
Mastalgia	0.25	0.16–0.40	< 0.001	0.56	0.34–0.92	0.02
Lump/palpable mass	7.05	3.74–13.29	< 0.001	10.57	4.89–22.84	< 0.001
Skin or nipple retraction	3.90	1.97–7.73	< 0.001	5.54	2.28–13.48	< 0.001
Age	1.07	1.05–1.08	< 0.001	1.07	1.05–1.08	< 0.001
Itch	0.77	0.10–5.75	0.80			
Erythema	0.89	0.21–3.74	0.87			
Nipple discharge	0.65	0.20–2.09	0.47			

## Discussion

In this population-based primary care study, we found that a palpable breast lump, skin or nipple retraction, and increasing age were strongly associated with higher odds of breast cancer. Mastalgia, when considered within a symptomatic primary care population, was associated with lower odds of breast cancer. Importantly, no cases of breast cancer were identified among women presenting with isolated mastalgia, regardless of whether the pain was localized or diffuse. It is important to note that these findings apply specifically to isolated mastalgia, defined as breast pain without any other breast-related symptoms at first presentation. Mastalgia occurring in combination with other symptoms, such as a palpable lump or skin changes, carries a higher risk of breast cancer and warrants careful assessment and appropriate diagnostic work-up.

Despite the very low likelihood of breast cancer in women with isolated mastalgia, GPs frequently initiated diagnostic imaging, often within three months of symptom onset. This occurred even though current Dutch primary care guidelines advise against routine imaging for isolated mastalgia of short duration [17]. These findings highlight a persistent gap between evidence-based recommendations and clinical practice.

### Comparison with existing literature

We found an incidence of breast-related complaints of 27.6 per 1000 patient years, which was in line with previous research from Eberl et al. (29.7 per 1000 patient years) [8]. The probability of breast cancer when consulting the GP solely for mastalgia was in line with previous research: we found 0% breast cancer in patients presenting with isolated mastalgia (0 of 588 patients), versus 0.4–3.2% in previous research [5]. The risk of breast cancer when presenting with a breast lump was consistent with previous studies: we found a 8.96% risk of breast cancer when consulting the GP for a lump, whereas previous research found a risk of 8.1% [8]. 

Compared with prior work, our study adds updated, symptom-based estimates derived entirely from primary care. Unlike studies that retrospectively identified patients based on a final diagnosis of breast cancer, we included all women presenting with breast-related symptoms and followed episodes forward to their final diagnosis. This design more closely reflects real-world diagnostic uncertainty faced by GPs at the time of first presentation.

### Implications for practice

Several factors may contribute to the high rate of imaging in women with isolated mastalgia. In our study, GPs documented abnormalities on physical examination in over one-third of cases that underwent imaging, most often described as probably benign glandular changes. Other unmeasured factors, such as patient anxiety, fear of missing a serious diagnosis, family history of breast cancer, or societal expectations regarding imaging as a reassurance tool, may also influence decision-making [18–21]. 

While diagnostic imaging can provide reassurance, it also carries potential harms, including false-positive findings, unnecessary follow-up investigations, increased patient anxiety, and higher healthcare costs [11, 22–25]. Mammography may be uncomfortable and exposes patients to radiation, further underscoring the importance of careful patient selection [25, 26]. 

Our results support current guideline recommendations that imaging and referral can often be safely deferred in women presenting with isolated mastalgia of short duration, provided there are no additional alarm symptoms [17]. Clear communication about the low likelihood of breast cancer in this subgroup may help GPs and patients engage in shared decision-making and reduce unnecessary diagnostic interventions.

Although healthcare systems differ internationally, the clinical presentation of breast-related symptoms in primary care is comparable, supporting the broader applicability of these findings beyond the Dutch setting.

### Strengths and limitations

A major strength of this study is the use of high-quality, routinely collected primary care data from a long-standing practice-based research network with comprehensive follow-up [13, 14]. The episode-based structure allowed us to link presenting symptoms, GP management decisions, and final diagnoses within the same clinical problem. By combining automated data extraction with detailed manual record review, we improved the accuracy of symptom classification and minimized misclassification. Another strength is the broad inclusion strategy. By including both women presenting with breast-related symptoms and all women ultimately diagnosed with breast cancer, we reduced the risk of missing atypical presentations and improved the generalizability of our findings to everyday primary care.

Several limitations should be considered. First, the number of episodes in women aged 76 years and older was relatively small (*n* = 66), limiting the precision of estimates in this age group. Second, although we distinguished between localized and diffuse mastalgia, we were unable to classify mastalgia as cyclic, non-cyclic, or extramammary, as has been done in previous literature [27]. Since 68% of patients reported symptoms lasting less than one month, such a classification was not feasible. Third, we did not analyze combinations of symptoms in detail, as this would have resulted in small subgroups and reduced statistical power. Fourth, physical examination findings were not included in the symptom variables used for regression analyses. As a result, some clinically relevant information influencing GP decision-making was not captured quantitatively. Fifth, we did not account for potential clustering at the level of patients, general practitioners, or practices in the statistical analyses. As a result, observations may not be fully independent, which could have led to underestimation of standard errors and overestimation of statistical significance. However, given the relatively low number of events per cluster and the exploratory nature of the study, we expect any resulting bias to be limited. Finally, patient-level factors such as health literacy, educational background, and individual cancer risk perception were not available, although these factors may influence both symptom presentation and diagnostic management.

## Conclusion

In this large primary care cohort, breast cancer was strongly associated with a palpable breast lump, skin or nipple retraction, and increasing age. Isolated mastalgia was not associated with breast cancer and did not result in any cancer diagnoses in our study population. However, mastalgia occurring in combination with other breast-related symptoms indicates a higher risk of breast cancer. Despite the low risk, diagnostic imaging was frequently performed in women with isolated mastalgia, often within three months of symptom onset. These findings provide updated, primary care–specific evidence to support guideline recommendations that imaging and referral may often be safely deferred in women presenting with isolated mastalgia of short duration. Incorporating this evidence into clinical conversations may help GPs reduce unnecessary investigations while maintaining safe and patient-centered care. Clinical decisions should continue to consider individual patient context and clinical judgment, balancing the potential benefits and harms of imaging.

## Data Availability

The datasets generated during and analysed during the current study are not publicly available due to traceable personal information within the dataset. Anonymized versions are available from the corresponding author on reasonable request.

## References

[1] Kim J, Harper A, McCormack V, Sung H, Houssami N, Morgan E, et al. Global patterns and trends in breast cancer incidence and mortality across 185 countries. Nat Med. 2025;31(4):1154–62. 39994475 10.1038/s41591-025-03502-3

[2] Netherlands Comprehensive Cancer Organisation (IKNL). [Available from: https://iknl.nl/borstkankercijfers. Accessed 05-01-2026

[3] Walker S, Hyde C, Hamilton W. Risk of breast cancer in symptomatic women in primary care: a case-control study using electronic records. Br J Gen Pract. 2014;64(629):e788–93. 25452544 10.3399/bjgp14X682873 PMC4240152

[4] Joyce DP, Alamiri J, Lowery AJ, Downey E, Ahmed A, McLaughlin R, et al. Breast clinic referrals: can mastalgia be managed in primary care? Ir J Med Sci. 2014;183(4):639–42. 24402166 10.1007/s11845-013-1066-z

[5] Martin-Diaz M, Maes-Carballo M, Khan KS, Bueno-Cavanillas A. To image or not in noncyclic breast pain? A systematic review. Curr Opin Obstet Gynecol. 2017;29(6):404–12. 28961632 10.1097/GCO.0000000000000407

[6] Dave RV, Bromley H, Taxiarchi VP, Camacho E, Chatterjee S, Barnes N, et al. No association between breast pain and breast cancer: a prospective cohort study of 10 830 symptomatic women presenting to a breast cancer diagnostic clinic. Br J Gen Pract. 2022;72(717):e234–43. 34990395 10.3399/BJGP.2021.0475 PMC8869188

[7] Koo MM, von Wagner C, Abel GA, McPhail S, Rubin GP, Lyratzopoulos G. Typical and atypical presenting symptoms of breast cancer and their associations with diagnostic intervals: Evidence from a national audit of cancer diagnosis. Cancer Epidemiol. 2017;48:140–6. 28549339 10.1016/j.canep.2017.04.010 PMC5482318

[8] Eberl MM, Phillips RL Jr., Lamberts H, Okkes I, Mahoney MC. Characterizing breast symptoms in family practice. Ann Fam Med. 2008;6(6):528–33. 19001305 10.1370/afm.905 PMC2582463

[9] Mohallem Fonseca M, Lamb LR, Verma R, Ogunkinle O, Seely JM. Breast pain and cancer: should we continue to work-up isolated breast pain? Breast Cancer Res Treat. 2019;177(3):619–27. 31309396 10.1007/s10549-019-05354-1

[10] Owen WA, Brazeal HA, Shaw HL, Lee MV, Appleton CM, Holley SO. Focal breast pain: imaging evaluation and outcomes. Clin Imaging. 2019;55:148–55. 30825809 10.1016/j.clinimag.2019.02.008

[11] Sivarajah R, Welkie J, Mack J, Casas RS, Paulishak M, Chetlen AL. A Review of Breast Pain: Causes, Imaging Recommendations, and Treatment. J Breast Imaging. 2020;2(2):101–11. 38424883 10.1093/jbi/wbz082

[12] Ader DN, Browne MW. Prevalence and impact of cyclic mastalgia in a United States clinic-based sample. Am J Obstet Gynecol. 1997;177(1):126–32. 9240595 10.1016/s0002-9378(97)70450-2

[13] Family Medicine Network. [Available from: https:/www.famenet.nl. Accessed 05-01-2026.

[14] Luijks H, van Boven K, Olde Hartman T, Uijen A, van Weel C, Schers H. Purposeful Incorporation of Patient Narratives in the Medical Record in the Netherlands. J Am Board Fam Med. 2021;34(4):709–23. 34312264 10.3122/jabfm.2021.04.200609

[15] International Classification of Primary Care, Second Edition (ICPC-2.) [Available from: https://www.who.int/standards/classifications/other-classifications/international-classification-of-primary-care. Accessed 05-01-20260.

[16] de Blok CJM, Wiepjes CM, Nota NM, van Engelen K, Adank MA, Dreijerink KMA, et al. Breast cancer risk in transgender people receiving hormone treatment: nationwide cohort study in the Netherlands. BMJ. 2019;365:l1652. 31088823 10.1136/bmj.l1652 PMC6515308

[17] De Bock GH, Bronsgeest MHE, Corsten MC, Hinloopen RJ, Korver JC, De Meij MA, et al. NHG-Standaard Borstkanker (derde herziening). Huisarts en Wetenschap. 2016;12:556–67.

[18] Salzman B, Collins E, Hersh L. Common Breast Problems. Am Fam Physician. 2019;99(8):505–14. 30990294

[19] Millet AV, Dirbas FM. Clinical management of breast pain: a review. Obstet Gynecol Surv. 2002;57(7):451–61. 12172222 10.1097/00006254-200207000-00022

[20] Altintas Y, Bayrak M. Evaluation of 1294 Female Patients with Breast Pain: A Retrospective Study. Adv Ther. 2018;35(9):1411–9. 30094702 10.1007/s12325-018-0769-y

[21] McCaul KD, Branstetter AD, Schroeder DM, Glasgow RE. What is the relationship between breast cancer risk and mammography screening? A meta-analytic review. Health Psychol. 1996;15(6):423–9. 8973921 10.1037//0278-6133.15.6.423

[22] Brodersen J, Siersma VD. Long-term psychosocial consequences of false-positive screening mammography. Ann Fam Med. 2013;11(2):106–15. 23508596 10.1370/afm.1466 PMC3601385

[23] Hafslund B, Nortvedt MW. Mammography screening from the perspective of quality of life: a review of the literature. Scand J Caring Sci. 2009;23(3):539–48. 19170959 10.1111/j.1471-6712.2008.00634.x

[24] Gram EG, Siersma V, Brodersen JB. Long-term psychosocial consequences of false-positive screening mammography: a cohort study with follow-up of 12–14 years in Denmark. BMJ Open. 2023;13(4):e072188. 37185642 10.1136/bmjopen-2023-072188 PMC10151842

[25] Neal CH, Helvie MA. Overdiagnosis and Risks of Breast Cancer Screening. Radiol Clin North Am. 2021;59(1):19–27. 33222997 10.1016/j.rcl.2020.09.005

[26] Armstrong K, Moye E, Williams S, Berlin JA, Reynolds EE. Screening mammography in women 40 to 49 years of age: a systematic review for the American College of Physicians. Ann Intern Med. 2007;146(7):516–26. 17404354 10.7326/0003-4819-146-7-200704030-00008

[27] Kataria K, Dhar A, Srivastava A, Kumar S, Goyal A. A systematic review of current understanding and management of mastalgia. Indian J Surg. 2014;76(3):217–22. 25177120 10.1007/s12262-013-0813-8 PMC4141056

